# Winner–loser effects overrule aggressiveness during the early stages of contests between pigs

**DOI:** 10.1038/s41598-020-69664-x

**Published:** 2020-08-07

**Authors:** Lucy Oldham, Irene Camerlink, Gareth Arnott, Andrea Doeschl-Wilson, Marianne Farish, Simon P. Turner

**Affiliations:** 1 Animal Behaviour and Welfare, Animal and Veterinary Sciences Department, Scotland’s Rural College (SRUC), West Mains Rd, Edinburgh, EH9 3JG UK; 2 Institute of Genetics and Animal Biotechnology, Polish Academy of Sciences, Ul. Postepu 36A, Jastrzebiec, 05-552 Magdalenka, Poland; 3 Institute for Global Food Security, School of Biological Sciences, Queen’s University Belfast, Belfast, BT9 7BL UK; 4 The Roslin Institute and R(D)SVS, University of Edinburgh, Easter Bush, Edinburgh, EH25 9RG UK

**Keywords:** Ecology, Psychology, Zoology

## Abstract

Contest behaviour, and in particular the propensity to attack an unfamiliar conspecific, is influenced by an individual’s aggressiveness, as well as by experience of winning and losing (so called ‘winner–loser effects’). Individuals vary in aggressiveness and susceptibility to winner–loser effects but the relationship between these drivers of contest behaviour has been poorly investigated. Here we hypothesise that the winner–loser effect on initiation of agonistic behaviour (display, non-damaging aggression, biting and mutual fighting) is influenced by aggressiveness. Pigs (n = 255) were assayed for aggressiveness (tendency to attack in resident−intruder tests) and then experienced two dyadic contests (age 10 and 13 weeks). Agonistic behaviour, up to reciprocal fighting, in contest 2 was compared between individuals of different aggressiveness in the RI test and experiences of victory or defeat in contest 1. Winner–loser effects were more influential than aggressiveness in determining initiation of agonistic behaviour. After accruing more skin lesions in contest 1, individuals were less likely to engage in escalated aggression in contest 2. The interaction between aggressiveness and winner–loser experience did not influence contest behaviour. The results suggest that aggressiveness does not compromise learning from recent contest experience and that reducing aggressiveness is unlikely to affect how animals experience winning and losing.

## Introduction

Across species, substantial variation in individuals’ reaction to unfamiliar conspecifics has been observed^1,2^. In resident−intruder tests, individuals are consistent in whether or not they attack an intruder across repeated tests^3,4^. Accordingly, aggressiveness can be viewed as a personality trait and is shaped by the genotype and early experience^5–8^. As well as inter-individual differences in aggressiveness, individuals modulate their own aggressive behaviour according to resource value^9^, estimation of own ability^10^, opponent characteristics^11^ and internal conditions influencing their evaluation of these factors (for example hormonal status^6^ or food deprivation^12^). There is also variation in how animals modulate their behaviour following victory or defeat in contests^13^, so called winner–loser effects. Aggressive contests are costly and it is important that animals learn from previous contest outcomes, not only about the identity of animals against which they have previously fought and won or lost^14^, but to extrapolate from past experience to estimate whether they are likely to be successful in future contests. Evidence suggests that aggressive animals are inflexible in modulating their behavioural strategy^15,16^ and we therefore hypothesise that highly aggressive animals are less likely to adapt their contest behaviour following victory or defeat than less aggressive animals.

Winning a contest increases the likelihood of winning a subsequent contest, and defeated individuals are more likely to lose again^17^. Even in hypothetical groups of equal resource holding potential (RHP, i.e. the individual’s fighting ability), dominance can be decided by winner and loser effects alone^18^. Furthermore, it is thought that *actual* fighting ability does not change, but rather that an animal re-evaluates its own competitive ability based on experience, and then up or down-regulates its aggressive behaviour^19^. Winner and loser effects are most relevant to the initial stages of a contest and once escalation occurs, intrinsic differences in RHP are more relevant to the outcome^19,20^. Both aggressiveness and winner–loser effects are potentially important drivers of contest behaviour, but we do not know how they interact. This study evaluates whether aggressiveness, in terms of a personality trait^21^, affects the ability to learn from recent victory or defeat, as determined by the strength of winner–loser effects on behaviour during the early stages of a subsequent contest.

Our study focusses on pigs (*Sus scrofa*), for which a validated method of assessing aggressiveness exists and where contest behaviour is easily stimulated and has been well described^22^. Pigs express individual differences in aggressiveness^3,23^ and their agonistic behaviour involves various stages of escalation starting from threat displays, to intense fights involving rapid and repeated biting^24^ that are associated with significant and measurable costs^25,26^. Aggressiveness in the resident−intruder test, in which a small intruder invades the territory of a resident, predicts the nature of aggressive behaviour expressed in dyadic contests in a novel environment^22^. Inter-individual differences in aggressiveness are influenced by early experience, such as litter size and sex ratio^27^. Furthermore, the individual differences in resident−intruder aggressiveness persist across group-mixing events^3^. Aggressiveness in pigs is an honest signal of willingness to initiate an agonistic encounter, while in contrast to some species^28^ it does not predict contest outcome^21^. In common with the male water skink *(Eulamprus quoyii*), initiation of aggression is less predictive of outcome in contests involving escalated fights^29^. In pigs, initiation of biting reliably predicts winning only in weight-matched contests that do not involve fighting^22^. Under these conditions, the initiator is almost always the most aggressive of the dyad.

There is evidence that aggressive animals are less flexible in their behaviour which, as tested in this study, may influence their susceptibility to winner–loser effects. Strains of highly aggressive mice show inflexibility in response to the changing environment compared to low aggressive strains^15^. Pigs categorised as having a “proactive” coping style due to their response to restraint at an early age have also been found to be less flexible in tests of reversal learning^16,30^ and are less likely to adapt their agonistic behaviour in line with their RHP^31^. Proactive pigs have been found to be more aggressive in a food competition test^32^ and a group mixing scenario^16^. In the context of group-mixing, differences in coping style may relate to the nature rather than the frequency of aggressive behaviour as one study found no difference in overall aggression between proactive and reactive pigs, but proactive pigs were more likely to continue fighting after dominance relationships had been established^31^.

Here, we consider the effects of victory and defeat in animals differing in aggressiveness on subsequent contest behaviour. We regard aggressiveness as “the tendency to attack an unfamiliar intruder in the resident−intruder test” whereas agonistic behaviour here refers to the display behaviour and aggression shown prior to a fight. We assess the effect of winning or losing a contest on two aspects of agonistic behaviour (i) initiation of agonistic behaviour and (ii) escalation of agonistic behaviour (towards mutual fighting). We predict that highly aggressive individuals will be less flexible (less likely to increase escalation following winning and decrease escalation following defeat) and be consistently highly aggressive (shorter latency and greater tendency to initiate agonistic behaviour) in a subsequent dyadic contest, whereas low aggressive winners will have a greater tendency and shorter latency to initiate agonistic behaviour than low aggressive losers. We predict that heavier pigs^22^ and males are more likely to be involved in fighting^27,33^ therefore we incorporate factors such as sex and body weight, which are likely to influence the outcome of the second contest, to account for the possibility that willingness to engage in aggressive behaviour in the second contest simply reflects a physical superiority in RHP.

## Methods

### Ethical statement

This study analysed existing behavioural data of 255 pigs in dyadic contests at 13 weeks of age, of which the methods have been described in detail by Camerlink et al.^34^. The experiment was approved by the UK Home Office and by the SRUC animal ethical review committee and was carried out in accordance with the relevant regulations and guidelines. The accommodation and care of the study animals adhered to the recommendations of the European Guidelines and UK Government (DEFRA) animal welfare codes. All experimental procedures were carried out under the guidance of SRUC’s named veterinary surgeon and adhered to the ASAB/ABS guidelines.

### Experimental design

**Figure 1:**
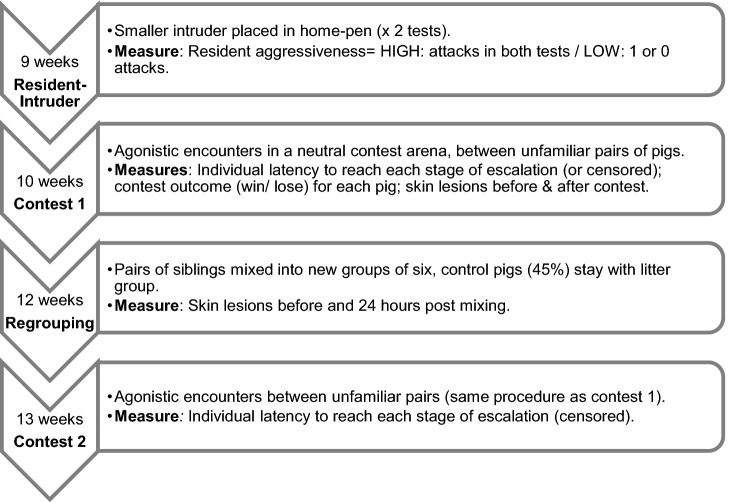
Timeline of experimental procedures, the age of the test pigs at each event and the behavioural measures analysed in the current study.

The relevant aspects of the procedures carried out by Camerlink et al*.*^34^ and the relevant measures used in the current study are briefly described in Fig. 1.

Animals were tested across four batches (i.e. four different birth cohorts), within which the treatments described below were balanced. A total of 311 pigs were housed in litter groups following weaning at 4 weeks of age. At 9 weeks of age they underwent two resident−intruder tests on consecutive days, to provide for each pig a measure of aggressiveness (described below). Dyadic contests between unfamiliar pigs were then staged at 10 and 13 weeks of age (also described below), hereafter referred to as C1 and C2.

Behaviour was recorded and observations were made using an established ethogram^21^ (Table 1). The sample we analysed included data from 255 pigs which completed two resident−intruder (RI) tests and experienced a conclusive winning or losing outcome in the first dyadic contest. Of the original 311, 22 pigs were excluded due to RI tests reaching premature end-points due to repeated mounting behaviour, intruder attacks or escape attempts, 19 pigs had an inconclusive C1 outcome, 8 pigs were excluded on both of these criteria and a further 7 pigs did not complete the study due to health reasons unrelated to the study (e.g. rectal prolapse).

**Table 1:** Ethogram of behaviour in contests; excerpt of behaviour relevant to the current study, from full list used in the larger study^34^.

Stage of escalation	Behaviour	Description
Display	Non-damaging investigation	Light touch with the nose or sniff body of other pig, without any forceful contact or biting
	Parallel walking	Pigs walk simultaneously with the shoulders next to each other with heads level
	Shoulder to shoulder	Standing or moving with the shoulder against the shoulder of the other pig whereby heads are frontal or parallel (not parallel walking) without putting real pressure on the shoulder
	Heads up	Both pigs have their nose lifted high up in the air alongside each other, either parallel or frontal
Non-damaging aggression	Pushing	Pig uses its head or shoulder to move the other pig aside while putting pressure on the shoulder
	Nose-wrestling	Pigs firmly press the side of their nose against the side of the nose of the opponent
	Head knock	Pig rapidly swings its head to deliver a blow
	Shove	Pig uses snout or head with continued pressure to move the other pig along or off the ground
	Flick	Pig uses small side nipping action of teeth or rapid but small sideways or upwards force of the head or snout that just touches the other pig without full biting
Biting	Biting	Pig opens its mouth and delivers a bite which contacts and injures the other pig
Mutual fight	Mutual fighting	Pig delivers a bite which is retaliated with an aggressive act from the opponent within five seconds and this continues until one pig retreats or until the pigs return to one of the other behaviours above
**End points**		
Contest win		One pig retreats without retaliation for one minute
Fear behaviour		Continuous attempts to escape for one minute (e.g. raising feet off the ground and against a wall, vocalizing loudly (screaming) continuously for 2 min, freezing behaviour in combination with hyperventilation lasting uninterrupted for 1 min)
Mounting		One pig lifts both front legs over the back, rear, side or head of another pig. Both front legs have to make contact over the other pig. Contest stopped if there are 5 full mounts, mounting lasts for 1 min continuously, or mounted animal becomes distressed (screaming, jumping) for one minute continuously or is at risk of injury

The study by Camerlink et al.^34^ focussed on the impact of regrouping on assessment strategies, and therefore at 12 weeks of age, 55% of the pigs experienced regrouping (see Fig. 1). The current study analyses the effect of individual differences in aggressiveness in the RI tests and experience of victory and defeat in C1, on the initiation of agonistic behaviour in C2. Although regrouping did not form part of our hypothesis, we considered that the more recent experience of winning or losing fights at regrouping might be relevant to C2 behaviour, so we included this treatment in the analysis. Since we expected that animals would have different experiences of regrouping aggression, but did not measure individual wins/losses, we used skin lesion scores (24 h post re-grouping) as an estimate of the physical costs incurred during fights after regrouping.

### Resident−intruder (RI) tests to measure aggressiveness

Habituation to social separation for the RI test and contests was carried out between the ages of 6–8 weeks, whereby pigs were habituated to the test location first in small groups, then in pairs, and finally individually, ensuring that these procedures were tolerated without overt fear responses. In the RI test, each individual resident was separated into one half of its home pen measuring 1.9 × 2.6 m , using a temporary, solid partition, and presented with an unfamiliar pig approximately 65% of the resident’s body weight, i.e. ‘the intruder’. Intruders did not act as residents (and vice versa) and were not used in subsequent contests. The intruders were additional to the 255 experimental pigs. Resident pigs experienced a different intruder on two consecutive test days. Pigs were categorised according to the number of tests in which they attacked the intruder (0, 1 or 2)^6,35^. Only pigs for which a robust measure of aggressiveness could be assigned were included. This excluded data for pigs when either test ended due to mounting, fear behaviour or attacks by the intruder (for details on end-points see Camerlink et al.^34^).

### Winner and loser experiences in contest 1

Winning and losing was determined by self-selection in dyadic contests at 10 weeks of age (C1). Contestants were matched into dyads with unfamiliar conspecifics and varied combinations of sex and body weight were included to allow assessment of these effects in the statistical models, i.e. to distinguish the effects of winning and losing from the effects of differences in RHP. The winner or loser status of the pigs depended on their performance in the contest, as opposed to creating artificial, “forced” winning and losing experiences, whereby pre-selected winner and loser test individuals are exposed to weaker or stronger opponents respectively^17^. The larger experiment by Camerlink et al.^34^, set out to examine assessment strategies in weight matched and asymmetric dyads, and therefore we consider the possible impact of weight difference. The asymmetric dyads may be considered to have had a “forced” winning or losing experience, whereas the weight matched dyads had a self-selected experience, so it is harder to separate the effects of differences in fighting ability and differences in C1 outcome on agonistic behaviour in C2. Nevertheless, the data from the weight matched dyads is of interest, firstly since fighting is more likely to be intense and prolonged between weight matched individuals^32^, and therefore the greater physical costs involved amplify the importance of learning from defeat, and secondly because the winner–loser effect may have most relevance in contests where initially animals cannot predict the outcome. Sex and body weight effects were nested such that half of C1 dyads were matched by body weight (mean percentage weight difference = 1.1 ± SD 1.2) and half were asymmetrical in weight (mean percentage weight difference = 20 ± SD 5.3). Within weight matched dyads, 48% were sex-mixed, 26% were female-female and 26% were male–male. Within weight asymmetric dyads, 44% were sex-mixed, 31% were female-female and 25% were male–male.

Contests were held in a neutral arena without any resources over which to compete. Both individuals were moved into the arena simultaneously and then the identity of the initiator and the latency of each pig to engage in each of four types of agonistic behaviour (display, non-damaging aggression, biting and mutual fighting) were recorded (see descriptions in Table 1). Contests ended when a clear winner emerged (one pig retreated without retaliation for one minute), when either animal demonstrated repeated fear behaviour or mounting or if the time limit of 30 min was reached (Table 1). A winner–loser experience in a neutral arena aims to recreate the experience of regrouping aggression, which usually takes place in a novel, neutral environment. In addition, contests between *K. Marmoratus* with restricted physical access demonstrate that conflict resolution is important for the development of winner–loser effects^36^.

The number of skin lesions (scratches from receiving bites) were counted in the morning before the contest and then immediately afterwards, as an indication of contest cost. Contests were video recorded to extract the latency and duration of involvement in each form of aggression.

### Group mixing

For the purposes of the larger study^34^, at 12 weeks of age 55% of pigs were mixed into groups comprising six pigs; a pair from each of three different litters. Controls remained with their siblings. Skin lesions were counted for all pigs pre- and 24 h post regrouping (or control treatment). The mixed pigs remained in their new group for the rest of the study.

### Contest 2

The same procedures were followed as for C1. At 13 weeks, unfamiliar pairs entered the same arena for a second contest, “C2”. In total, 53% of C2 dyads were matched by body weight (mean percentage weight difference 2.9 ± SD 2.3) and the remaining 47% had a mean percentage weight difference of 22 ± SD 6.9. Forty-eight percent of C2 dyads were sex-mixed, 27% were female-female and 25% were male–male. In 21% of C2 dyads, both contestants were C1 winners, in 19% they were both losers and the remaining 60% of dyads comprised one winner and one loser. Mean body weight of prior winners was 57 kg (SD 7 kg) and of losers 54 kg (SD 7 kg). Each dyad was selected from the same treatment; regrouped or control.

### Selection criteria for data used in the statistical analyses

Two main effects were investigated, each with two levels: aggressiveness (number of times each pig attacked an intruder in RI tests, with two levels) and C1 outcome (win/lose). Agg+ represents test pigs that attacked the intruder in both tests and Agg− indicates that they attacked the intruder in only one or neither test. Inclusion criteria for this study were: (i) completion of both resident−intruder tests without reaching a premature endpoint, and (ii) a conclusive winning or losing experience in C1. After applying these criteria, 255 out of 311 pigs were retained in the dataset.

### Outcome variables

The early contest period was defined as up to and including the initiation of mutual fighting. The stages of escalation during this period (see Table 1) were defined as display behaviour (non-damaging investigation, parallel walking, adopting shoulder to shoulder or “heads up” positions), non-damaging aggression (pushing, nose-wrestling, head-knock, shove or flick), biting and mutual fighting (focal pig delivers a bite, which is retaliated within 5 s). These stages were ranked from low-risk forms of investigation and threat, to high-risk escalated attack behaviour. Where an initiator could not be assigned for a behaviour because the contestants were observed to reach this stage of escalation simultaneously, an initiator was assigned at random (using the random function in Excel).

The outcome measures were:

(i) Latency to initiate each stage of escalation in C2
(ii) Highest level of escalation reached in C2 compared with C1
(iii) Initiation of agonistic behaviour in C2

### Statistical models

Statistical tests used to evaluate the above outcomes were (i) Linear mixed models for continuous traits, (ii) Multinomial models for categorical traits and (iii) Generalised linear mixed models for categorical (binomial) traits. All analyses were carried out in “R” version 3.5.1^37^.

(i) Latency to initiate agonistic behaviour in contest 2.

Linear mixed models using restricted maximum likelihood (package “lmerTest”)^38^ were used to evaluate the main effects of C1 outcome, RI aggressiveness and their interaction, on the initiation latency of each stage of escalation in C2 separately: display, non-damaging aggression, biting and mutual fighting. To avoid pseudo-replication in the analyses described below, only initiators were compared (regarded as the focal pig), resulting in one data point for each dyad and the non-initiating pig was described as the “opponent”. Fixed effects considered in the full models were: the C1 outcomes (win/lose) of the focal pig and of their C2 opponent, the RI aggressiveness of the focal pig and C2 opponent, the body weight of the focal pig (kg), the relative body weight difference between focal and C2 opponent (% difference) and the interaction between these two effects, the sex of the focal individual and their C2 opponent, the regrouping treatment of the C2 dyad, the body lesions accumulated by the focal pig during regrouping (or the same period of time for control pigs) and the body lesions accumulated by the focal pig during C1. Additionally, we included the interactions between focal C1 outcome and focal C1 body lesions, between C1 outcome of focal and opponent pigs and between focal pig aggressiveness and focal pig C1 outcome. The regrouping treatment that occurred for 55% of the pigs between C1 and C2 did not form part of the hypothesis but fights between unfamiliar pigs in-between contests may have overshadowed the effect of C1 on C2 behaviour, so regrouping treatment and lesion counts during the initial 24 h post-regrouping (or control) were included as an estimate of regrouping aggression. Batch (i.e. birth cohort) and litter (i.e. sibling group) of the focal and C2 opponent individuals were included as nested random effects (litter nested within batch).

The residuals of the full models were first checked by inspecting residual plots for normality and heterogeneity of variance, and then using formal tests of normality (Shapiro–Wilk test). Where the data were not normally distributed, the outcome variables were natural-log transformed (latency to initiate display and latency to initiate biting) after which they satisfied model assumptions. C1 total lesion counts and regrouping lesion counts differed significantly from a Gaussian distribution (Anderson–Darling test) even when transformed and so were categorised as below or above median values, which were 29 lesions during C1 and 28 lesions during the 24 h post-regrouping, or the equivalent period for control animals.

To determine the statistically significant influencing effects on early C2 behaviour, the “drop1” function in R was used to obtain the partial F-test result for single term deletions. Fixed effects and interaction terms with *p *values greater than 0.1 were removed from the model, after which all terms were evaluated again (to allow evaluation of terms involved in interactions in the global model as main effects). At every stage of reduction, Wald tests (“anova” function in “car” package)^39^ were applied with significance level *p* < 0.05, to evaluate the change in goodness of fit and to identify the most parsimonious model of best fit. The relative contribution of fixed effects to the variance in latency to initiate agonistic behaviour were extracted from the linear models using “lsmeansLT”^38^, and post-hoc pairwise comparisons within interactions and categorical independent variables were carried out using Tukey’s adjusted test. Transformed outcome variables were reported as back-transformed LS means with associated confidence levels, calculated from back-transforming the least-square mean of transformed data (m), m − S.E. and m + S.E. respectively.

(ii) Escalation of aggression by individuals in contest 2, compared with their contest 1 behaviour.

A multinomial model (package “nnet”)^40^ was used to determine whether C1 outcome, aggressiveness, and their interaction influenced whether the stage of escalation each individual reached in C2 was the same, higher or lower than in C1. Firstly, each individual was scored from 1 to 5 according to the level of escalation they reached in each contest (see Table 1), representing either no agonistic behaviour, display, non-damaging aggression, biting or mutual fighting respectively. The outcome variable was defined as “increase escalation” (C2 > C1), “same level of escalation” (C2 = C1) or “decrease escalation” (C2 < C1). In the multinomial model, the reference outcome was defined as “same level of escalation”. Fixed effects considered in the full model were the same as for the linear models described above, with the addition of the relative body weight of C1 compared to C2 opponents (% difference) and the sex of both C1 and C2 opponents. The full model was rationalised by removing non-significant terms as for the linear models. The least-squared means of each fixed effect were extracted from the model and post-hoc pairwise comparisons within interactions and categorical independent variables were carried out using Tukey’s adjusted test. Transformed outcome variables were reported as back-transformed LS means with associated confidence levels, calculated from back-transforming the least-square mean of transformed data (m), m − S.E. and m + S.E. respectively.

Since the model used (“multinom”) did not allow inclusion of random effects, the model output was compared with that of two separate generalised linear mixed effects models (GLMM) (glmer, package “lme4”)^41^. These separate models assessed a) individuals’ tendency to increase escalation over the two contests, and b) individuals’ tendency to decrease escalation, using a binomial link-function. The random effects in the GLMM were the litter nested within batch of the focal individual and of their C1 and C2 opponents. The binomial models identified the same significantly influential fixed effects and the direction of their effects, as the multinomial model. The multinomial model output is reported here, as it better represents the three possible outcomes: increasing escalation, decreasing escalation or reaching the same level of escalation in both contests.

(iii) Tendency to initiate agonistic behaviour in contest 2.

For each stage of escalation, the GLMM method, using a binomial link-function, was used to test whether initiating a stage of escalation in C1 influenced the likelihood of initiating the same stage of escalation in C2. The models also assessed whether the tendency to initiate agonistic behaviour in C2 was influenced by the interaction between initiating in C1 and C1 outcome (e.g. whether those animals which initiated agonistic behaviour in C1 and then won were more likely to initiate again in C2 than those that had initiated and lost). Fixed and random effects were as for the linear mixed models, with the additional main effect “initiated stage of escalation in C1”, which could be 1 (yes) or 0 (no), and the interaction between initiation of agonistic behaviour in C1 and C1 outcome.

## Results

### Resident−intruder aggressiveness

Of 311 residents, 175 attacked the intruder in both tests, 75 attacked in one test and 33 did not attack the intruder in either test. The remaining 28 individuals had at least one test result which reached a premature endpoint. Due to the low number of non-attackers, the two categories “attack once” and “no attacks” were combined to form a category “Agg−”.

### Contest 1 (winning and losing experiences)

There were ten contests that reached an end-point due to a fear response or mounting, and five finished with an undetermined outcome^34^. The larger opponent won in 59% of asymmetric C1 dyads overall. If contestants were sex-matched, then the larger pig won on 70% of occasions. Almost half (48%) of males which were smaller than their opponent won the contest, compared with only 18% of smaller females. In weight-matched, mixed sex contests, males won on the majority of occasions (76%), as compared to females (19%) whilst 5% were undecided.

After applying inclusion criteria, our data set for analysis of C2 behaviour included 129 C1 losers, of which 59% were categorised as Agg + (based on RI testing) and 126 C1 winners, of which 68% were in the category Agg+. In total, 139 pigs were from the regrouping treatment and 116 were controls. Of the female pigs used in C2, 59% were categorised as Agg+ and of the male pigs, 68% were Agg+.

(i) Latency to initiate agonistic behaviour in contest 2.

Variables that did not significantly influence the latency to initiate any stage of escalation were the absolute weight of the focal pig, the relative weight difference between C2 dyadic contestants, the cost of C1 (lesions), the cost of regrouping (lesions) and the RI aggressiveness of the C2 opponent.

### Effect of contest 1 outcome and aggressiveness

**Figure 2:**
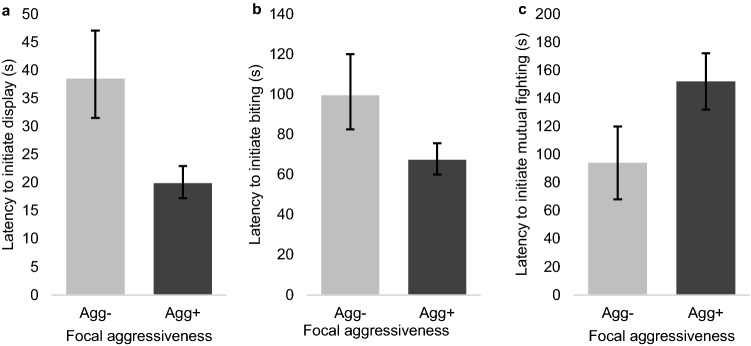
The effect of aggressiveness on latency to initiate agonistic behaviour in contest 2. (**a**) Latency to initiate display, (**b**) latency to initiate biting and (**c**) latency to initiate mutual fighting. Results of "display" and "biting" models are represented as back-transformed least-square means, with lower and upper confidence intervals calculated from back-transforming the least-square mean of transformed data (m), m − S.E. and m + S.E. respectively.

Winners of C1 went on to initiate agonistic behaviour faster than C1 losers across all stages of escalation in C2, except for non-damaging aggression, where there was no difference in latency between C1 winners and losers (as shown in Table 2). Pigs that attacked in both resident−intruder tests (Agg+) were on average faster to initiate display behaviour in C2 (Fig. 2) and Agg+ pigs also had a non-significant tendency to be faster to initiate biting (Fig. 2). In contrast, Agg+ pigs were slower to initiate mutual fights. Differences in latency to initiate non-damaging aggression between Agg+ and Agg− pigs did not reach statistical significance (*p* > 0.1). The interaction between focal C1 outcome and aggressiveness did not affect the latency to perform agonistic behaviours in C2.

**Table 2:** The effect of aggressiveness and C1 outcome and other factors on the latency to initiate agonistic behaviour in contest 2.

Outcome variable	Predictor variable	DF	F	*p* value	LS means and confidence intervals (s)
Latency to initiate display (log_e_-transformed)	Focal contest 1 outcome	74	7.9	0.006	
	Win				21 (18–24)
	Lose				37 (31–44)
	Focal aggressiveness	82	9.2	0.003	
	Agg+				20 (17–23)
	Agg−				38 (31–47)
	Regrouping treatment	74	4.0	0.048	
	Regrouped				34 (29–40)
	Control				23 (19–27)
Latency to initiate non-damaging aggression	Focal contest 1 outcome	39	0.56	0.46	
	Win				67 (54–85)
	Lose				83 (64–107)
	Focal aggressiveness	37	0.14	0.71	
	Agg+				71 (57–87)
	Agg−				79 (60–104)
Latency to initiate biting (log_e_-transformed)	Focal contest 1 outcome	86	8.4	0.005	
	Win				59 (51–67)
	Lose				105 (90–122)
	Focal aggressiveness	86	3.7	0.057	
	Agg+				67 (60–76)
	Agg−				99 (83–120)
	Opponent contest 1 outcome	86	8.4	0.005	
	Win				62 (53–71)
	Lose				99 (85–116)
	Sex contest 2 dyad (focal-opponent)	86	12	< 0.001	
	Female–Female				38 (32–46)^a^
	Female–Male				77 (62–97)^ab^
	Male–Female				86 (68–108)^b^
	Male–Male				159 (132–192)^b^
Latency to initiate mutual fighting	Focal contest 1 outcome	37	5.7	0.022	
	Win				97 (75- 119)
	Lose				150 (128- 172)
	Focal aggressiveness	40	5.5	0.024	
	Agg+				152 (132- 172)
	Agg−				94 (68- 120)
	Opponent contest 1 outcome	35	8.6	0.006	
	Win				89 (67–111)
	Lose				158 (135–181)
	Sex contest 2 dyad (focal-opponent)	38	7.9	< 0.001	
	Female–Female				45 (18–72)^a^
	Female–Male				111 (81–141)^b^
	Male–Female				156 (120–192)^b^
	Male–Male				180 (157–203)^b^

Data are presented as back-transformed least-square means, accompanied by lower and upper confidence intervals, calculated from back-transforming the least-square mean of transformed data (m), m − S.E. and m + S.E. respectively. Values with different superscript letters differed by *p* < 0.05 in post-hoc analysis.

### Contest 1 outcome of the opponent

Biting and mutual fighting were initiated sooner in C2 if the opponent (i.e. the non-initiating pig) was a winner in the previous contest, C1 (see Table 2). Opponent C1 outcome did not influence latency to initiate lower stages of escalation (display and non-damaging aggression).

### Sex of the contestants

The sex of dyadic contestants was a significant factor in the initiation of biting and fighting (see Table 2). Female pigs initiated biting twice as fast if the opponent was female than if their opponent was male. Males did not significantly differ in their latency to first bite according to the sex of their opponent. Within sex-mixed dyads, males and females took a similar length of time to initiate biting. Of the sex-matched dyads, female-female dyads were on average four times faster to reach the “biting” stage of escalation than male–male dyads. Latency to initiate mutual fighting followed the same pattern, with females initiating fights against another female in less than half the time of any other sex combination. The greatest contrast was between female-female and male–male dyads. For the lower stages of escalation (display and non-damaging aggression), sex of the contestants did not influence initiation latency and was excluded from the models.

### Regrouping treatment

Animals that experienced regrouping at 12 weeks were on average slower to initiate display in C2 than control animals. Regrouping experience did not influence the latency to initiate any later stage of escalation.

(ii) Escalation of aggression by individuals in C2, compared with their C1 behaviour.

Overall, 48 individuals (19%) reached a higher stage of escalation in C2 than C1, 90 (35%) maintained the same level of escalation and 117 (46%) reduced their level of escalation between C1 and C2. As main effects, individual aggressiveness and C1 outcome did not influence the tendency to increase or decrease escalation from C1 to C2 (see Table 3). However, animals that received few (below median) lesions in C1 were substantially more likely to increase escalation, compared with animals which had received a high number of lesions (above median) in C1. There was a significant interaction effect between focal and opponent C1 outcome on the likelihood of decreasing escalation from C1 to C2, compared with the likelihood of reaching the same level of escalation in both contests (see Table 3).

**Table 3:** The effect of contest outcome, aggressiveness and other factors on the likelihood of decreasing or increasing escalation across contests, compared to their likelihood of reaching the same maximum stage of escalation in both contests.

Predictor variable (baseline value)	Decrease escalation from Contest 1 to Contest 2		Increase escalation from contest 1 to contest 2	
	Odds ratio (confidence interval)	*p* value	Odds ratio (confidence interval)	*p* value
Focal contest 1 outcome (Lose)				
Win	1.3 (0.81–2.1)	0.57	0.43 (0.23–0.81)	0.18
Focal aggressiveness (Agg−)				
Agg+	0.91 (0.67–1.3)	0.88	1.1 (0.67–1.6)	0.90
Opponent contest 1 outcome (Lose)				
Win	2.2 (1.4–3.6)	0.081	1.1 (0.60–2.0)	0.88
Focal contest 1 lesions (Low)				
High	1.1 (0.76–1.5)	0.83	**0.27 (0.13–0.47)**	**0.0040**
Contest 1 outcome: focal-opponent (Lose–Lose)				
Win–Lose	1.5 (1.1–2.0)	0.14	0.69 (0.49–0.99)	0.30
Lose–Win	**2.0 (1.5–2.6)**	**0.014**	1.2 (0.82–1.6)	0.67
Win–Win	**0.56 (0.46–0.70)**	**0.0063**	0.89 (0.69–1.1)	0.61
Sex of contest 2 dyad: focal-opponent (Female-Female)				
Female–Male	1.5 (0.98–2.4)	0.35	0.95 (0.52–1.7)	0.94
Male–Female	1.6 (1.01–2.43)	0.31	0.58 (0.29–1.2)	0.44
Male–Male	**0.20 (0.13–0.32)**	** < 0.001**	0.99 (0.58–1.7)	0.98

Odds-ratios were calculated by back-transforming model coefficients (log-odds) accompanied by lower and upper confidence intervals, calculated from back-transforming the model coefficient (b), b − S.E. and b + S.E. respectively. A 2-tailed Z test was applied post-hoc to the multinomial model (Z = log-odds/ standard error). Values in bold were significant at *p* < 0.05.

Focal losers paired with a prior winner were twice as likely to decrease escalation than if they were paired against a prior loser and focal animals in dyads composed of two winners were significantly less likely to decrease escalation from C1 to C2 than focal animals in dyads composed of two losers. There was no significant difference between any combination of focal and opponent C1 outcome on the likelihood of increasing escalation.

The sex of both C2 contestants was an important factor in whether individuals reduced their escalation in C2 compared with C1 but not in whether they increased escalation. Where the focal and opponent in C2 were both female, the focal individual was 5 times more likely to reduce escalation, compared with C2 dyads where both contestants were male. Mixed sex contestants did not significantly differ in their behaviour from those in female-female contests (see Table 3).

(iii) Tendency to initiate agonistic behaviour in C2.

Winners of C1 were more likely than losers of C1 to initiate display behaviour in C2 (odds ratio: 2.1, CI 1.6–2.8, *p* = 0.007), and biting in C2 (odds ratio: 2.0, CI 1.5–2.6, *p* = 0.014). Of the total sample of C2 contestants with known RI-aggressiveness and C1 outcome, 21% initiated display in C2 after winning C1 and 13% initiated display in C2 after having lost C1. Twenty-three percent of pigs won C1 and then initiated biting in C2, whereas only 17% initiated biting in C2 after being defeated in C1. There was no evidence for a relationship between C1 outcome and tendency to initiate non-damaging aggression or fighting in C2. We found no evidence that the animals that initiated any stage of escalation in C1 had a significantly higher tendency to initiate the same stage of escalation again in C2. RI aggressiveness did not significantly influence an individual’s tendency to initiate any stage of escalation in C2, nor did the interaction between aggressiveness and C1 outcome. The sex of both contestants in C2 had an interaction effect on their tendency to initiate biting and fighting. Post-hoc pairwise comparisons using Tukey’s adjusted test showed that on average, females tended to be more likely than males to initiate biting against a female opponent (odds ratio 2.7, CI 1.8–4.0, *p* = 0.058). Similarly, the likelihood of male pigs initiating a fight in C2 was on average five times higher if paired with a male than with a female opponent (odds ratio 0.19, CI 0.12–0.32, *p* = 0.0061).

## Discussion

Aggressiveness, as a personality trait, and prior contest experience both independently influenced behaviour in a subsequent contest. We assessed the interaction of both factors and did not find that aggressiveness modulates winner–loser experience, in contrast to our expectation that high aggressiveness would limit behavioural flexibility. Contest experience and aggressiveness were both more influential than body weight (as a proxy measure of RHP) on pigs’ behaviour in the early contest period. Here we demonstrate uniquely enduring winner–loser effects, over a three-week interval and regardless of exposure to aggression during social mixing between contests.

### Effects of contest outcome and aggressiveness

Winner–loser effects are anticipated in pigs, since previous studies have shown that they become more aggressive after being allowed to dominate an unfamiliar pig^23,42^, are sensitive to social defeat^43^ and modify their behaviour to avoid future attacks when paired with an individual which has recently defeated them^14^. Our results confirm the existence of winner–loser effects in pigs, winners of a dyadic contest (C1) behaved more aggressively than losers in the early stages of a subsequent contest (C2), with both a greater tendency and shorter latency to initiate agonistic behaviours. In addition to contest outcome, the physical cost incurred in C1 influenced how pigs adapted their behaviour in C2. After receiving a high number of skin lesions in C1, individuals were much less likely to increase the level of escalation, compared with those that received fewer lesions. We considered skin lesions a reliable indicator of contest cost, since in this experiment they correlated strongly with mutual fight duration and changes in blood glucose and lactate pre to post contest (which were sampled and analysed as part of the preceding study)^34^. Winners and losers of C1 diverged in their latency and tendency to initiate agonistic behaviour in C2 but there was no evidence that individuals which initiated certain stages of escalation in C1 were more influenced by the effects of winning and losing than those which did not initiate in C1. In an experimental study^44^, sticklebacks (*G*. *aculeatus*) were rewarded for initiating display behaviour towards an opponent (the experimenters removed the opponent, simulating contest success), which resulted in a greater tendency to perform threat displays. A preceding study did not succeed in teaching sticklebacks to cease display behaviour using punishment^45^. This illustrates the complexity of contest behaviour. The effects of victory and defeat cause animals to adapt their contest strategy, but this adjustment does not follow a simple “initiate or do not initiate” rule which is either reinforced by winning or punished by losing. Instead, pigs exhibited a general increase or decrease in aggression depending on the overall adversity of the contest experience.

Higher aggressiveness did not influence the likelihood of initiating any stage of escalation, in agreement with prior findings that aggressiveness does not influence likelihood of escalated fighting^22^ but was strongly associated with faster initiation of display behaviour and tended to result in faster initiation of biting. On the contrary, higher aggressiveness was associated with slower initiation of mutual fighting in C2, suggesting that individual differences in aggressiveness have specific effects depending upon the level of escalation. The RI aggressiveness of the opponent did not significantly influence latency and tendency to initiate agonistic behaviour, suggesting that initiation of agonistic encounters is predominantly controlled by individual traits of the focal animal, and the motivation to initiate agonistic contact is not dependent on how willing the opponent is to engage.

Aggressiveness did not reduce the impact of winning and losing on subsequent early contest behaviour. In *K. marmoratus,* aggressiveness corresponds to high levels of cortisol, testosterone and keto-testosterone, and individuals with these hormonal profiles were found to be less susceptible to winner–loser effects^46^. Whilst aggressiveness in pigs is linked to greater adrenal weight and cortisol levels^47^, the results of this study do not support a relationship between aggressiveness and response to contest outcome, contrary to our prediction that aggressiveness would reduce flexibility. Here, we considered the response to C1 outcome as a potential indication of individual behavioural flexibility. In contrast with tests of flexibility such as reversal learning in a T-maze^16^, the consequence of an “incorrect” decision in C1 (i.e. involvement in aggression which ends in a physically costly defeat) may be more profound than the absence of a food reward, and could have overshadowed subtle differences in cognitive ability between pigs of different aggressiveness. Furthermore, it can be argued that attacking an intruder in the home pen in the resident−intruder test is adaptive and demonstrates social competence^48^. Therefore a more complete definition of “highly aggressive personality” may need to be developed, for example including a propensity towards excessive or abnormal aggression^49^ such as persistence despite the submission of an opponent^50^.

### Persistence of winner–loser effects

The influence of a single winning or losing experience on subsequent aggressive behaviour is typically short-lived and winner–loser effects on contest behaviour have been found to disappear between 60 min and four days after a contest in a range of insect, fish, reptile, bird and rodent species^20,51,52^, although the effects of repeated social defeat are often cumulative^53,54^, and past experience of defeat can modulate winner–loser effects^55^. The winner–loser effects on contest behaviour found in our study were much more persistent and were evident even after experience of formation of a new social group. A single social defeat has been shown to influence heart rate 10 days later^56^ and behaviour in an open field test four weeks later in rodents^57^, but to our knowledge, the effects of a single win or defeat on contest behaviour have not been demonstrated across this time period.

Most recent experience usually dominates in decision making and this is true of animals undergoing multiple contests^17^. We found that the individuals that had experienced group mixing between contests were slower to initiate display behaviour in C2, but the regrouping treatment and its associated physical cost did not influence any other measure of C2 behaviour. In accordance with the effects on assessment strategy previously analysed^34^, the impact of C1 outcome was influential despite the intervening social experience. This indicates that information gained in C1 was specifically relevant to the context of a dyadic contest, or that the dyadic contest had more profound psychological or physiological effects than agonistic encounters in the group setting. Indeed, the average number of skin lesions incurred during the brief (up to 30 min) contest was comparable to the median number of skin lesions pigs accrued across a 24-h period of social interactions following regrouping. Contrary to the theory that species displaying frequent social interactions would retain information from past encounters to a lesser extent^13^, the effects of winning and losing appeared to persist for 3 weeks even in this highly social species. Since the test environment was only used for contests and was different from the home pen, the experience of winning or defeat may have been linked with these environmental cues, which supports evidence that context is important to winner and loser effects^58^. Although we incorporated a proxy-measure of involvement in aggression during the 24 h post-regrouping, since this is when aggression peaks, we did not assess dominance indices. Furthermore, although we subtracted the number of lesions present immediately before C2 from those present afterwards, we did not model the effect of having a large or small number of pre-existing lesions on subsequent contest behaviour. Work in K. *marmoratus* suggests that recent fighting experience can amplify the effects of losing a contest one month previously^55^.

### Dominance of loser over winner effects

Winner–loser effects on pigs’ latency and tendency to initiate agonistic behaviour in C2 were evident, but as we compared winners with losers instead of winners and losers with contest-naïve animals, the relative importance of winning or losing was not directly tested. When we compared escalation in C1 (using naïve contestants) with escalation in C2 (after winning or losing) we found that the interaction between focal and opponent C1 outcome influenced likelihood of reducing but not likelihood of increasing the maximum level of escalation in which they engaged. Specifically, when faced with a C2 opponent who had won its first contest, a focal animal that had lost C1 was more likely to decrease escalation than one that had won. In crickets, losing was also more influential than winning on the likelihood of escalating to physical contact in a subsequent contest^59^. On the contrary, winning enhanced tendency to initiate attacks in *K. marmoratus*^19^ more than losing decreased this tendency. For young animals such as those in this study, losing should have a higher influence than winning, since it is adaptive for young animals to be maximally aggressive and gain information about their own RHP by fighting and then reducing aggression as their true RHP becomes clear^47^. When pigs are mixed into unfamiliar groups, the centralisation of aggression within a social network increases with age, such that between groups of unfamiliar newly weaned pigs, a higher proportion of possible dyads will fight^60^, compared with pigs approaching puberty^61^ and adult sows^60,62^. This study advances our understanding of why certain individuals become more aggressive with age, despite the reduction in the proportion of the population choosing to engage in fighting. The consequences of aggression become more severe with age and body weight, and accordingly we found that the number of pigs which engaged in fighting reduced from C1 to C2, and this inter-contest interval coincided with a period of rapid growth (10 to 13 weeks of age). However, the individual differences in aggressive personality established in early life did not fully predict those individuals which were faster and more likely to initiate agonistic behaviour in C2. Therefore, we conclude that learning from contest experience has an important influence on future agonistic behaviour.

### Effects of body weight and sex on early contest 2 behaviour

The effects of prior contest experience and aggressiveness on early contest behaviour (initiating and escalating contests) dominated over the influence of contestants’ absolute and relative body weights, echoing findings in hens^63^ and in fish^64^. There is some evidence that pigs cannot assess fighting ability without physical contact^14^ and therefore even against a larger opponent, they choose to engage in early contest behaviour in order to gain more reliable information^19^. Confinement in the contest arena may also have influenced contest strategies,weaker individuals had limited opportunity to retreat and therefore might have used attack as a form of defence. The absolute and relative RHP of opponents influences later stages of a contest, such as persistence in mutual fighting^29^ (for assessment strategies used in the full contests of the current study, see^34^), although contest outcome is weakly predicted by body weight in pigs^65^ and fighting style can also be influenced by neurophysiological differences^66^. Contest decisions were highly impacted by the sex of the C2 contestants, but not by a contrast between the C1 and C2 opponent sex or size, implying that winner–loser effects are generalised to future contests^19^ rather than associated with specific characteristics of the opponent. By 13 weeks of age, male pigs appear to attach a higher value to defeating other males than females, which may reflect the ecological role of aggression in competition for mates in this species^67^.

### Effect of opponent contest 1 outcome

Focal animals initiated damaging aggression more quickly against a prior winner than against a prior loser. Opponent C1 outcome did not influence the actual tendency of focal individuals to initiate agonistic behaviour or not, unlike *K. marmoratus*^19^, where focal individuals were less likely to initiate attacks against prior winners. However, the combined C1 outcome of both contestants influenced how likely pigs were to decrease escalation following their C1 experience. These opponent effects indicate that within a dyad, the presence of a prior winner drives the contest towards escalation, regardless of which pig out of the dyad initiates agonistic behaviour.

## Conclusion

We found that winner–loser effects are important and long-lasting in pigs. Although aggressiveness as a trait which develops in early life has an influence on how willing individuals are to initiate agonistic behaviour in contests, social experience can override this phenotypic predisposition. We found no evidence that more aggressive animals are less susceptible to winner–loser effects on their subsequent expression of aggression.

## Data Availability

The data that support the findings of this study are available upon request from the corresponding author, L.O.
